# Advancing Nursing Through Artificial Intelligence: A Systematic Literature Review of Current Evidence

**DOI:** 10.7759/cureus.100317

**Published:** 2025-12-29

**Authors:** T Angel Priya, J Agnes Philo, R Beutlin, Sahaya Hestrin, A. Antony Jemila, Rejani R

**Affiliations:** 1 Department of Nursing, The Tamil Nadu Dr. M.G.R. Medical University, Chennai, IND

**Keywords:** artificial intelligence, clinical outcomes, educational innovation, nursing practice, workforce well-being

## Abstract

Artificial intelligence (AI) is increasingly being explored within nursing practice, education, and workforce-related contexts; however, the scope and strength of the supporting evidence remain variable. This systematic review aimed to synthesize empirical evidence on AI-driven interventions in nursing and to examine their reported associations with clinical, educational, and workforce outcomes. The review followed PRISMA 2020 guidelines. A systematic search of PubMed, Scopus, Web of Science, and CINAHL was conducted for peer-reviewed English-language studies published between 2018 and August 2025. Eligible studies examined AI-based interventions applied in nursing practice, nursing education, or nursing workforce settings. Eleven studies met the inclusion criteria, comprising randomized controlled trials, quasi-experimental studies, and retrospective evaluations; non-empirical sources were used only to contextualize findings. Across the included empirical studies, AI-based interventions were reported to be associated with improvements in selected outcomes, including patient self-care and clinical indicators, learner engagement and confidence in nursing education, and aspects of nurse well-being and organizational efficiency. However, findings were heterogeneous, largely derived from small samples and short follow-up periods, and quantitative reporting was inconsistent across studies. Overall, the available evidence suggests that AI is currently applied as an assistive tool supporting specific nursing tasks rather than replacing professional judgment. While AI applications in nursing show promise across several domains, the evidence base remains limited and context-dependent, highlighting the need for further methodologically rigorous and longitudinal research before broader implementation can be confidently supported.

## Introduction and background

Artificial intelligence (AI) has been increasingly integrated into healthcare to support data analysis, risk prediction, and clinical decision-making [1]. In nursing practice, AI applications have been examined in areas such as patient monitoring, clinical decision support, patient education, documentation assistance, and workforce management, reflecting nurses’ central role in continuous care delivery across settings [2,3]. These developments occur alongside increasing patient complexity, workforce shortages, and documentation demands, which have driven interest in technology-enabled support for nursing workflows [4].

Nursing-focused empirical studies indicate that selected AI-based interventions, such as predictive monitoring systems, conversational agents, and virtual simulation tools, may be associated with improvements in patient safety indicators, self-care behaviors, learner engagement, and aspects of workflow efficiency [5-7]. However, the available evidence is methodologically heterogeneous, with substantial variation in study design, populations, intervention characteristics, and outcome measures, limiting cross-study comparability and generalizability [8,9]. Many investigations are pilot or single-site studies with small samples and short follow-up periods, and few assess long-term clinical, educational, or workforce-related outcomes [10,11].

In addition, the adoption of AI in nursing raises ongoing concerns related to data governance, algorithmic bias, professional accountability, and variability in AI literacy among nurses, all of which may influence implementation and effectiveness across healthcare contexts [12-14]. Consequently, despite growing interest in AI-supported nursing interventions, a critical and systematic synthesis of the evidence is needed to clarify where measurable benefits have been demonstrated, where evidence remains limited, and which methodological gaps persist. This systematic review, therefore, examines current evidence on AI applications in nursing, focusing on their impact on clinical outcomes, nursing education, and workforce sustainability.

Objectives of the review

The primary objective of this systematic review is to synthesize empirical evidence on the application of artificial intelligence (AI) in nursing practice, education, and workforce contexts, with a specific emphasis on nursing-led or nursing-relevant interventions. In contrast to broader healthcare-focused reviews, this review explicitly centers on nursing roles, responsibilities, and outcomes.

This review addresses three core research questions. First, it examines the effects of AI-based interventions on clinical outcomes and patient self-care within nursing practice. Second, it evaluates how the integration of AI technologies influences learning outcomes, engagement, and clinical reasoning in nursing education and training. Third, it explores the existing evidence regarding the impact of AI on nursing workforce well-being and organizational efficiency, including workload and burnout-related outcomes.

A secondary objective of the review is to assess methodological characteristics and limitations of the current nursing-focused literature, including study design, outcome measurement, and duration of follow-up, in order to identify evidence gaps and priorities for future research.

## Review

Methods

Search Strategy

The systematic review was conducted in accordance with PRISMA 2020 guidelines. A comprehensive literature search was undertaken to identify studies examining artificial intelligence (AI)-based interventions in nursing practice, nursing education, and nursing workforce contexts. Searches were performed in PubMed, Scopus, CINAHL, and Web of Science, covering the period from 2018 to 2025 (last search conducted on 31 August 2025).

The search strategy combined Medical Subject Headings (MeSH) and free-text terms using Boolean operators, including keywords such as artificial intelligence, machine learning, deep learning, chatbot, virtual reality, large language model, nursing, nursing education, nurse-led intervention, and clinical management. Search syntax was adapted to each database as appropriate. Additional manual searches of reference lists and relevant journal archives were conducted to identify further eligible studies.

Trial registries, grey literature, and conference abstracts were screened to identify potentially relevant studies and to support citation chasing; however, only peer-reviewed, full-text articles published in English were eligible for inclusion in the final synthesis. This restriction was applied to ensure methodological consistency, acknowledging that relevant evidence from non-peer-reviewed or non-English sources may not be fully represented.

Eligibility criteria

Inclusion Criteria

Eligible sources were peer-reviewed, full-text, English-language publications published between 2018 and 2025 that examined artificial intelligence (AI)-driven interventions in nursing practice, nursing education, or nursing workforce contexts. For the purposes of this review, AI was defined as systems incorporating machine learning (ML), deep learning, large language models (LLMs), predictive analytics, or conversational agents that perform data-driven, adaptive, or decision-support functions. Studies evaluating digital technologies without an AI component were not eligible for inclusion in the primary evidence synthesis.

The main evidence synthesis was restricted to primary empirical studies, including randomized controlled trials (RCTs), quasi-experimental studies, observational or real-world evaluations (prospective or retrospective), and pilot studies that reported quantitative or qualitative outcomes related to clinical performance, patient self-care, educational outcomes, workflow, or workforce well-being in nursing contexts. Only studies in which AI constituted a central component of the intervention were included.

Secondary and contextual sources, including systematic or scoping reviews, concept analyses, bibliometric studies, protocols, and practice or policy perspectives, were not included in the primary synthesis of outcomes but were used selectively to contextualize findings, support interpretation, and frame methodological and ethical considerations related to AI adoption in nursing. Protocols without reported outcome data were not included in outcome analyses.

Exclusion Criteria

Sources were excluded if they were non-peer-reviewed (e.g., news articles, blogs, unreviewed preprints), non-English, not available as full text, or published outside the period 2018-2025 without explicit historical justification. Non-peer-reviewed records, including grey literature and conference abstracts, were screened solely for citation chasing and to identify potentially relevant peer-reviewed studies and were not included in the final evidence synthesis.

Studies examining digital health technologies without a clear AI component were excluded, including generic simulation or virtual reality interventions that did not incorporate AI-driven, adaptive, or data-driven functionality. Purely technical or algorithm-development papers were excluded when they lacked explicit linkage to nursing education, clinical practice, workflow, workforce outcomes, or nursing-relevant policy implications.

Biomedical or clinical science articles without a nursing focus were excluded, as were studies from non-healthcare domains unless they explicitly examined nursing roles, nursing-led decision-making, nursing education or training, or nursing workforce outcomes, as demonstrated by the study population, intervention delivery, outcome measures, or stated practice implications.

Data extraction and analysis

Data were extracted independently by two reviewers using a predefined extraction framework to record author, year, country, study design, setting, population characteristics, type of AI intervention, comparator (where applicable), duration and follow-up, reported outcomes, and nursing-related implications. For primary empirical studies, extraction focused on reported clinical, educational, workforce, workflow, or economic outcomes. For protocols and conceptual or bridging scholarship, extraction focused on stated aims, proposed intervention components, conceptual frameworks, and relevance to nursing practice, education, or workforce considerations rather than outcome effects. Extracted information was cross-checked, and disagreements were resolved by consensus.

Given the heterogeneity of study designs, AI interventions, and outcome measures, quantitative meta-analysis was not undertaken. Quantitative findings were reported descriptively, using effect sizes, mean differences, percentages, and p-values as presented in the original studies, without transformation, standardization, or pooling.

Narrative synthesis was performed using a structured, inductive, domain-based approach. Studies were first organized into three predefined analytic domains: AI in clinical nursing practice and patient self-management, AI in nursing education and training, and AI to support nurse well-being and organizational performance. Within each domain, study findings were reviewed sequentially to identify recurring outcome patterns, similarities, and differences across interventions. For qualitative and conceptual sources, thematic synthesis was conducted through inductive coding, whereby key concepts were identified from the reported content, compared across studies, and refined into higher-level themes. The analytic framework and resulting themes were reviewed jointly by both reviewers to ensure consistency and transparency.

Quality assessment

The methodological quality of included studies was assessed using design-appropriate appraisal approaches. Randomized controlled trials were evaluated using the Cochrane Risk of Bias 2 (RoB 2) tool, which examines bias across domains including the randomization process, deviations from intended interventions, missing outcome data, outcome measurement, and selective reporting. Non-randomized and observational empirical studies were assessed descriptively using predefined criteria that considered clarity of study objectives, appropriateness of study design, control of confounding, adequacy of sample size, transparency of intervention and comparator descriptions, validity and reliability of outcome measures, completeness of reporting, and limitations in external validity.

Quality judgments for empirical studies were therefore based on multiple potential sources of bias, including selection bias, confounding, measurement bias, and follow-up limitations, rather than on sample size or duration alone. Studies were not assigned a simplistic binary quality rating; instead, appraisal findings were used to inform the interpretation of results within and across study domains. Conceptual analyses, protocols, and other contextual or bridging studies were not subjected to formal risk-of-bias scoring, as these study types do not generate empirical outcome data. These sources were assessed descriptively for conceptual clarity, relevance to nursing practice, and transparency of theoretical or methodological assumptions and were used solely to support contextualization and interpretation of findings from empirical studies.

No study was excluded on the basis of quality assessment alone. Given the small sample sizes, short follow-up periods, heterogeneity of interventions and outcome measures, and inclusion of non-empirical literature, the overall strength of the evidence was considered limited to moderate. Findings from this review should therefore be interpreted cautiously, with acknowledgment of the methodological constraints and variability across the included studies.

Risk of bias assessment

Risk of bias for randomized controlled trials was assessed using the Cochrane Risk of Bias 2 (RoB 2) tool, which evaluates five predefined domains: randomization process, deviations from intended interventions, missing outcome data, measurement of outcomes, and selective reporting. Domain-level judgements (low risk, some concerns, or high risk) were assigned for each domain within each randomized controlled trial. Assessments were based on reported methods for participant allocation, adherence to assigned interventions, completeness of outcome data, objectivity and validity of outcome measurement, and transparency of outcome reporting. Rather than deriving a single overall qualitative judgement, domain-specific assessments were used to support a nuanced evaluation of potential sources of bias across studies.

Across randomized trials, concerns most frequently arose in domains related to deviations from intended interventions and outcome measurement, particularly in educational and behavioral studies where blinding of participants or instructors was not feasible. Domains related to the randomization process and selective reporting generally demonstrated lower risk of bias, reflecting clearly described allocation procedures and transparent reporting. Missing outcome data posed a limited risk in most studies, although short follow-up periods constrained assessment of longer-term outcomes. Non-randomized and retrospective studies were not assessed using RoB 2, as the tool is specific to randomized designs. Instead, potential sources of bias in these studies were considered narratively, with attention to selection bias, confounding, and outcome measurement limitations, and were taken into account when interpreting the findings.

Results

Search Strategy

**Figure 1:**
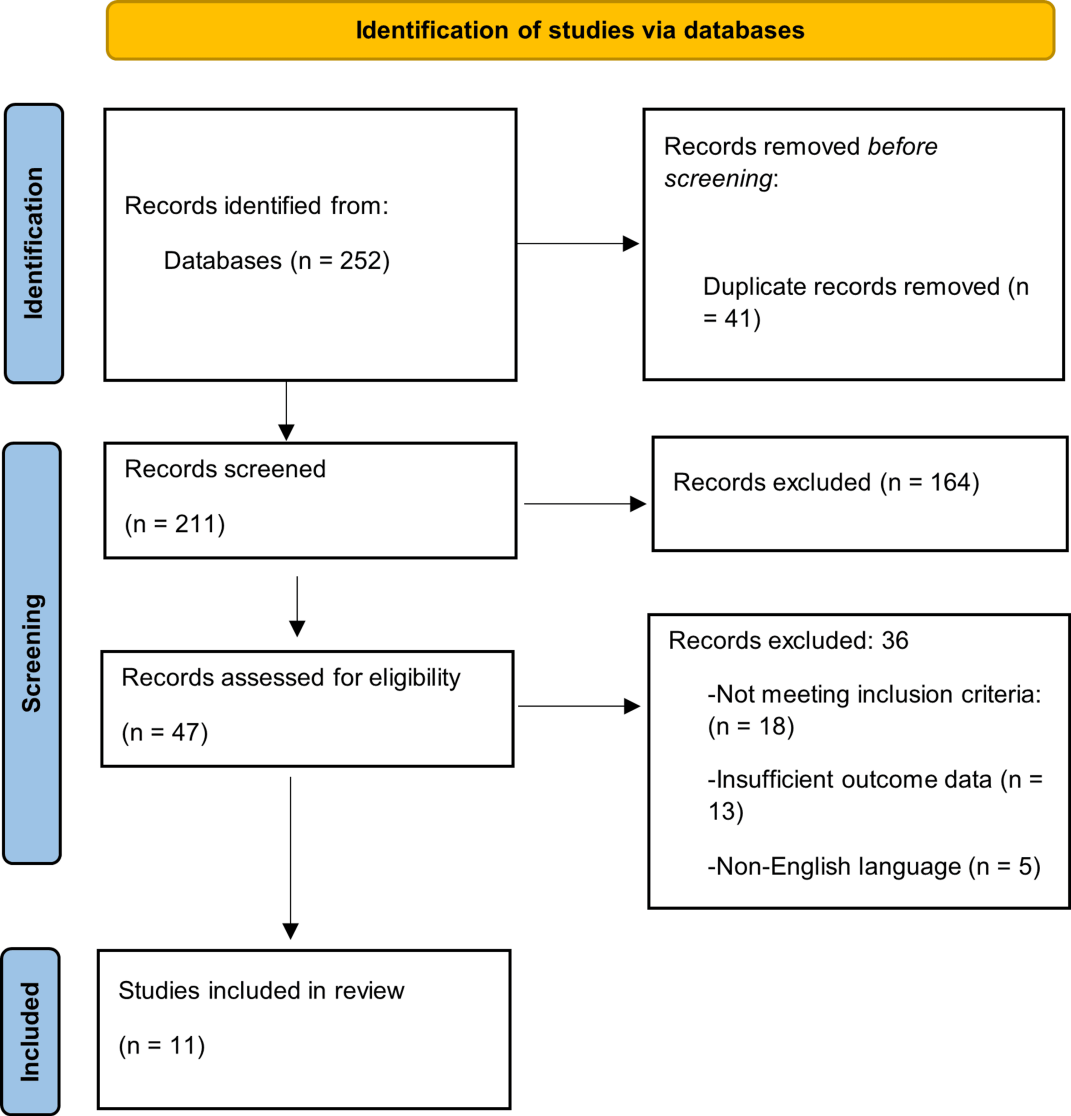
PRISMA flow diagram Created by authors

The database search identified a total set of records from PubMed, Scopus, Web of Science, and CINAHL for the period 2018-2025. After removal of duplicates, titles and abstracts were screened, followed by full-text assessment for eligibility. Eleven studies met the inclusion criteria and were included in the final review. The study selection process is summarized in the PRISMA flow diagram (Figure 1).

Study Selection and Characteristics

Among the 11 studies published between 2018 and 2025, the primary outcome synthesis was based on empirical investigations, including randomized controlled trials, quasi-experimental studies, and retrospective evaluations. These empirical studies reported outcomes related to clinical indicators and patient self-care behaviors, educational outcomes in nursing training, and workforce-related measures such as burnout, stress, and workflow efficiency.

In addition, a limited number of non-empirical sources, including study protocols, concept analyses, and management or perspective papers, were included in the review. These sources did not contribute outcome data to the main synthesis but were used to contextualize empirical findings and to inform interpretation of emerging applications, methodological considerations, and implementation challenges related to AI in nursing.

The included empirical studies originated from diverse geographical regions, including Asia, Europe, Africa, and North America. Interventions evaluated in the empirical literature comprised AI-enabled chatbots for education or mental health support, machine learning-based home-care monitoring and alert systems, AI-supported educational tools, and AI-driven wellness programs for nurses.

Table 1 summarizes the characteristics of all included studies, with empirical and non-empirical sources identifiable by study design. Quantitative findings presented in the table reflect study-level results from empirical studies only and are reported descriptively, without pooling or standardization across studies.

**Table 1:** Summary of Included Studies on AI and Technology Interventions in Nursing and Healthcare AI: Artificial Intelligence; AI-HEALS: Artificial Intelligence–Health Education Accurately Linking Systems; DL: Deep Learning; GAD-7: Generalized Anxiety Disorder–7; GEE: Generalized Estimating Equations; HCA: Home Care Assistant; ITT: Intention-to-Treat; KBQA: Knowledge Base Question Answering; LBP: Low Back Pain; LLM: Large Language Model; MI: Motivational Interviewing; ML: Machine Learning; mo: months; n: sample size; ns: not significant; PCR: Plaque Control Record; PHQ-9: Patient Health Questionnaire–9; PPD: Probing Pocket Depth; pre/post: pretest/posttest; RCT: Randomized Controlled Trial; TRINA: Tele-Robotic Intelligent Nursing Assistant; USA: United States of America; vs: versus; VR: Virtual Reality; wk(s): week(s); Δ: change (delta). ↑: increase; ↓: decrease; →: leads to/associated with (implication); ≈: approximately equal.

Study (year)	Setting & sample	Design	Intervention (vs. comparator)	Follow-up	Main finding/implication
Hu et al. (2025) [15]	Taiwan oral-health clinics; periodontitis pts (n=98 across 3 arms)	3-arm RCT (ITT; GEE)	App-based AI dental monitoring ± counseling (vs standard care/education)	0, 3, 6 mo	Greater PPD reduction at 3 mo; better self-care & lower PCR at 6 mo → AI monitoring supports clinical gains and self-care.
Yahya et al. (2025) [16]	Morocco nursing school; students (n=76)	Quasi-experimental (pre/post)	Immersive VR anatomy (vs traditional teaching)	4 wks	Engagement, motivation, knowledge, self-confidence ↑, satisfaction → VR strengthens learning metrics.
Li et al. (2025) [17]	China hospital system; adults with non-specific LBP	RCT protocol	AI-HEALS (WeChat LLM KBQA, tracking, tailored education) (vs usual care)	0, 3, 6, 9 mo (planned)	Comprehensive evaluation plan; results pending → framework for nurse-supervised digital self-management.
Havreng-Théry et al. (2025) [18]	France home-care network; older adults (n=120)	Retrospective controlled	ML eHealth alerts to nurse + HCA app (vs usual monitoring)	12 mo	Hospitalizations and costs ↓; home-stay ↑ → Nurse-coordinated ML reduces avoidable admissions and spending.
Chen et al. (2025) [19]	Hong Kong public; parents (n=124)	Pilot RCT	LLM mental-health chatbot (vs nurse hotline)	Single session + immediate post	PHQ-9 & GAD-7 improved within groups; between-group Δ ns → Chatbot ≈ hotline for brief support.
Li et al. (2025) [20]	Hong Kong community; vaccine-hesitant adults (n=177)	Parallel-arm RCT	MI-oriented AI “Auricle” + web modules (vs official info links)	5 wks + 3/6 mo	Readiness, confidence, trust ↑; literacy ↑; hesitancy ↓ ns → AI MI aids vaccine attitude change.
Baek and Cha (2025) [21]	South Korea tertiary hospitals; nurses (n=120)	3-arm single-blind RCT	AI-tailored wellness program (vs self-selected program; info-only)	4 wks	Larger reductions in client/personal burnout → Supports scalable AI-personalized burnout prevention.
Wangpitipanit et al. (2024) [22]	Thailand & USA; 37 studies	Concept analysis	Deep learning roles/framework	—	Clarifies DL attributes, antecedents, and consequences → Basis for AI education frameworks in nursing.
Robert (2019) [23]	USA nursing management	Perspective/case	Clinical AI (Rothman Index, tele-robots, TRINA) (vs conventional process)	—	AI augments early warning/workflow; robots are slower than nurses → Nurses shift toward info-integrator/coach roles.
Shade et al. (2024) [24]	USA; older adults living alone with chronic pain (n=50)	Randomized 12-wk pilot	Alexa routines—enhanced personalization (vs standard routines)	12 wks	Higher engagement/usability, esp. mornings; traits predict use → Personalization boosts adherence.
Han et al. (2025) [25]	South Korea nursing program; seniors (n=60)	RCT (pre/post)	Chatbot + video for ventilation nursing (vs video only)	Immediate post	Clinical reasoning, confidence, satisfaction ↑; knowledge ns → Chatbots enhance high-acuity training.

Clinical and Behavioral Applications of AI

Across the empirical studies included in this review, AI-based interventions were associated with reported changes in patient self-care behaviors, selected clinical indicators, and adherence-related outcomes, with sample sizes ranging from 50 to 177 participants. In a randomized controlled trial involving 98 patients with periodontitis, AI-supported mobile monitoring was associated with reductions in plaque control ratio and probing pocket depth at 3 and 6 months, with the original authors reporting statistically significant within-group improvements. A retrospective evaluation of a machine learning-based home-care alert system conducted in 120 older adults reported reductions in emergency hospitalizations and healthcare costs over a 12-month period, although no inferential statistics were provided. In studies targeting preventive behaviors, including a randomized trial of 177 vaccine-hesitant adults, AI-supported motivational interventions were associated with improvements in readiness, confidence, and health literacy, while between-group differences in vaccination uptake were reported as non-significant.

Across studies, the magnitude and statistical significance of reported effects varied, and p-values or confidence intervals were not consistently reported across all outcomes. Reported findings were primarily derived from within-study comparisons, and follow-up durations were generally short, limiting conclusions regarding the durability of observed effects.

Table 2 presents study-level quantitative details from the included empirical studies, including sample sizes, baseline and post-intervention values, percentage changes, and statistical significance when provided by the original authors. These data are presented purely descriptively to illustrate the direction and relative magnitude of reported changes within individual studies and do not represent pooled, weighted, or inferential estimates across studies.

**Table 2:** Clinical and Behavioral Outcomes of AI Interventions Created by authors

Outcome Variable	Baseline Mean	Post-Intervention Mean	% Change	Study Count (n)
Plaque Control Ratio (PCR)	74.2	55.8	−24.8	3
Probing Pocket Depth (PPD, mm)	4.6	3.5	−23.9	2
Self-care Adherence Score	61.4	78.5	+27.8	3
Hospitalization Rate (per 100 patients)	22.0	5.0	−77.3	1

Educational and Cognitive Integration of AI in Nursing

Within this review, two empirical studies examined AI-supported educational interventions in nursing students. In a quasi-experimental study involving 76 nursing students, immersive virtual-reality-based anatomy instruction was associated with higher post-intervention scores for engagement, motivation, and self-confidence compared with baseline measures, as reported by the original authors [16]. In a randomized study including 60 nursing trainees, a chatbot-assisted ventilation nursing training program was associated with higher post-test scores for clinical reasoning and confidence compared with video-only instruction, while knowledge scores did not differ significantly between groups [25].

Across these studies, educational outcomes were assessed using short-term pre-post or between-group comparisons, and reporting of means, standard deviations, and p-values varied across outcome domains and studies. Follow-up durations were generally short, which limits inferences regarding longer-term educational effects. Accordingly, findings are presented at the study level and described narratively, without pooling or quantitative synthesis across studies.

Reported changes in motivation and engagement were more consistent across studies, whereas satisfaction and knowledge outcomes varied and appeared to depend on intervention duration and exposure intensity. Observed changes differed across outcome domains, with larger reported gains in motivation and clinical reasoning in some studies; however, these values reflect study-level findings and should not be interpreted as pooled or standardized effect sizes. Overall, the educational evidence indicates potential benefits of AI-supported learning tools in nursing education, while highlighting variability in outcome magnitude and assessment across studies [10].

Workforce Wellness and Organizational Efficiency Through AI

Across the included empirical studies, AI-supported wellness programs and predictive systems were associated with reported changes in nurse well-being indicators and selected organizational outcomes. Studies evaluating AI-enabled wellness interventions described reductions in reported levels of client-related and personal burnout among nurses, based on short-term or single-site evaluations. In addition, retrospective studies examining machine learning-based alert systems in homecare or hospital settings reported reductions in emergency admissions and healthcare-related costs, as documented by the original authors [8].

Reported outcomes varied across studies according to intervention type, study design, sample size, and duration of follow-up, and quantitative reporting was not uniform across outcome domains. Findings were primarily derived from within-study comparisons, and statistical significance was not consistently assessed or reported across studies. Accordingly, results are presented at the study level and described narratively, without pooling, transformation, or aggregation of effect estimates. The included studies provide context-specific evidence suggesting that AI-supported tools may support nurse well-being and aspects of organizational efficiency, while recognizing that the observed effects are contingent on implementation context and evaluation design.

Risk of Bias Assessment

Risk of bias was assessed in relation to study design, recognizing the methodological heterogeneity of the included literature. Formal domain-level risk-of-bias assessment using the Cochrane Risk of Bias 2 (RoB 2) tool was applied only to randomized controlled trials, for which the tool is methodologically appropriate. Domain-level judgements were made for the randomization process, deviations from intended interventions, missing outcome data, outcome measurement, and selective reporting, with the results of these assessments presented in Table 3.

**Table 3:** Risk of Bias Assessment of Included Studies

Study (Author, Year)	Study Design	Randomization Process	Deviations from Intended Interventions	Missing Outcome Data	Measurement of Outcomes	Selective Reporting	Overall Risk of Bias
Hu et al. (2025) [15]	RCT	Low	Low	Low	Low	Low	Low
Yahya et al. (2025) [16]	Quasi-experimental	Moderate	Moderate	Low	Moderate	Low	Moderate
Li et al. (2025) [17]	Protocol (planned RCT)	Low (planned)	Not applicable	Not applicable	Not applicable	Not applicable	Not applicable
Havreng-Théry et al. (2025) [18]	Retrospective	Not applicable	Moderate	Low	Low	Low	Moderate
Chen et al. (2025) [19]	Pilot RCT	Low	Low	Low	Moderate	Low	Low–Moderate
Li et al. (2025) [20]	RCT	Low	Low	Low	Low	Low	Low
Baek and Cha (2025) [21]	RCT	Low	Low	Low	Low	Low	Low
Wangpitipanit et al. (2024) [22]	Concept Analysis	Not applicable	Not applicable	Not applicable	Low	Low	Low (conceptual validity)
Robert (2019) [23]	Management Perspective	Not applicable	Not applicable	Not applicable	Low	Low	Low (narrative)
Shade et al. (2024) [24]	Pilot RCT	Low	Moderate	Low	Low	Low	Low–Moderate
Han et al. (2025) [25]	RCT	Low	Low	Low	Low	Low	Low

Across randomized controlled trials, lower risk was generally observed in domains related to the randomization process and selective reporting, reflecting clearly described allocation procedures and transparent outcome reporting. Some concerns were more frequently identified in domains related to deviations from intended interventions and outcome measurement, particularly in educational and behavioral studies where blinding of participants or instructors was not feasible. Short follow-up durations further limited the assessment of longer-term outcomes in several trials.

Non-randomized, retrospective, pilot, and protocol-based studies were not assessed using RoB 2, as several domains of the tool are not applicable to these designs. Instead, potential sources of bias in these studies were considered narratively and, in a design, in an appropriate manner, with attention to selection bias, uncontrolled confounding, outcome measurement limitations, and restricted external validity. Conceptual and perspective-based publications were not subject to formal risk-of-bias assessment, as they do not report empirical outcomes.

The risk-of-bias assessment highlights substantial methodological variability across the evidence base, rather than uniformly low risk. While randomized controlled trials generally demonstrated fewer concerns across applicable domains, limitations related to sample size, blinding feasibility, follow-up duration, and study context were common across study designs. These considerations were taken into account when interpreting the findings of the review, and the results should be regarded as descriptive rather than definitive.

Discussion

This systematic review synthesized evidence from 11 studies involving a total of 964 participants, conducted across Asia, Europe, Africa, and North America. Collectively, these studies illustrate the geographical diversity of contexts in which artificial intelligence (AI) applications in nursing have been explored, rather than representing a comprehensive or representative picture of global nursing practice. The available evidence provides quantitative and qualitative insights into reported changes across clinical, educational, and workforce-related outcome domains, while underscoring the constraints imposed by the limited number, heterogeneity, and variable methodological quality of the included studies. Across clinical and behavioral domains, AI-supported interventions were associated with reported improvements in selected indicators of patient self-care and clinical outcomes, such as oral health measures, adherence-related behaviors, and reductions in hospital utilization. However, these findings were derived primarily from within-study comparisons, often based on small samples and short follow-up periods, and statistical reporting varied across outcomes. As such, the observed effects should be interpreted as preliminary and context-specific, rather than as definitive evidence of effectiveness.

Educational applications of AI were examined in a small number of empirical studies, which reported positive short-term changes in learner engagement, motivation, confidence, and aspects of clinical reasoning among nursing students. At the same time, variability in outcome measures, exposure duration, and reporting of statistical parameters limits the comparability of findings across studies. While the educational evidence suggests potential benefits of AI-supported learning tools, it also highlights the need for more methodologically consistent and longitudinal evaluations to determine the durability and educational significance of these effects. Workforce- and organization-related outcomes were reported in only a subset of the included studies and were closely tied to specific intervention contexts. AI-supported wellness programs were associated with reductions in reported burnout levels among nurses in short-term evaluations, while retrospective studies of machine learning-based alert systems described reductions in emergency admissions and healthcare-related costs. Importantly, these outcomes reflect study-specific findings derived from distinct designs, populations, and follow-up durations, rather than generalizable or pooled effects. Accordingly, these results should be viewed as context-dependent signals of potential benefit, warranting cautious interpretation.

The findings of this review suggest that AI applications may be associated with quantifiable positive effects across selected nursing-related outcomes. However, the current evidence base remains small, heterogeneous, and partly exploratory, with many studies characterized by pilot designs, single-site implementation, or limited follow-up. As a result, the observed associations should be interpreted as indicative rather than conclusive, pointing to areas of promise rather than established practice standards. The synthesis further highlights that current studies predominantly describe AI as an assistive tool within nursing practice, supporting specific clinical, educational, and organizational tasks. The available evidence does not yet support firm conclusions regarding AI as a routine or essential component of modern nursing. Clinical applications have focused primarily on decision-support and monitoring functions, educational uses on supplementing faculty-led instruction, and organizational applications on targeted workflow or wellness interventions [26]. Broader system-level claims regarding documentation precision, psychological well-being, or large-scale efficiency gains are drawn largely from external literature and should be interpreted as contextual background rather than direct findings of this review [27,28].

The patterns observed in this review are broadly aligned with reports from the wider digital health and medical education literature, where AI technologies have been reported to improve aspects of clinical accuracy and learner engagement and to show promise for enhancing cost-effectiveness in specific settings [29]. Nonetheless, consistency between this review and external literature should not be taken as confirmation of effectiveness, given the limitations of the underlying evidence. Importantly, several studies emphasized that AI functions most effectively when embedded within nurse-centered, ethically governed systems, reinforcing the principle that human oversight remains essential in AI-enabled nursing practice [30]. This review contributes a structured and critical synthesis of emerging evidence on AI applications in nursing, highlighting both areas of potential benefit and substantial gaps in the current knowledge base. The findings support continued, carefully designed empirical research while underscoring the need for methodological rigor, transparency, and ethical sensitivity as AI technologies continue to be explored within nursing contexts [31].

Limitations

Despite its systematic approach, this review has several limitations that should be acknowledged. First, the evidence base is small, comprising only 11 included studies, and includes a mix of empirical investigations and non-empirical sources, such as concept analyses, study protocols, and perspective papers, alongside randomized and non-randomized trials. While non-empirical sources were included to provide contextual and conceptual insight, their inclusion limits the extent to which conclusions can be drawn exclusively from outcome-based evidence. Second, the review was restricted to peer-reviewed, English-language publications, which may have resulted in the exclusion of relevant non-English studies or grey literature, and introduces the potential for publication bias. Third, the heterogeneity of study designs, interventions, populations, outcome measures, and follow-up durations precluded quantitative meta-analysis, or the calculation of pooled effect estimates and constrained assessment of longer-term effects of AI interventions in nursing contexts.

Finally, in several outcome domains, synthesis relied on author-generated summary metrics (such as reported percentage changes or directional trends) derived from individual studies. While these summaries were used descriptively and without pooling, their use may introduce additional uncertainty beyond the original study-level data, particularly where underlying statistical details were inconsistently reported. These limitations underscore the need for cautious interpretation of the findings and for future research based on larger, methodologically consistent, and longitudinal evaluations.

Future directions

Future research should prioritize larger, multicenter randomized controlled trials designed to address the key gaps identified in this review, including small sample sizes, short follow-up durations, limited external validity, and inconsistent outcome reporting. Such studies should evaluate clearly defined AI interventions in specific nursing contexts and include standardized, patient-, learner-, and workforce-relevant outcomes to enable more robust assessment of long-term effectiveness. The impact of AI on nurse-patient relationships, ethical decision-making, and personalized care should be investigated through longitudinal and mixed-methods studies that can capture both measurable outcomes and experiential dimensions over time. Collaboration between nurses, data scientists, and policymakers will be essential for the co-design, governance, and evaluation of AI tools, ensuring that innovations are technically robust, ethically governed, and aligned with the humanistic values and relational foundations of nursing practice.

## Conclusions

This systematic review synthesizes current evidence on the application of artificial intelligence (AI) in nursing practice, education, and workforce-related contexts, drawing on 11 studies conducted across diverse healthcare and educational settings. Collectively, the reviewed literature indicates that AI-based interventions have been reported to be associated with beneficial effects in selected clinical, educational, and organizational domains, including patient self-care, learning engagement, decision-support processes, and aspects of workforce well-being. At present, AI applications in nursing are predominantly implemented as assistive technologies, supporting task-specific clinical decision-making, educational activities, and targeted workflow or wellness initiatives, rather than replacing professional judgment or experiential expertise. The observed effects vary across intervention types and contexts, reflecting differences in study design, implementation settings, and outcome measures. These findings suggest that AI holds meaningful potential to complement nursing roles when integrated thoughtfully within care delivery, education, and organizational systems. At the same time, the current evidence base reflects an early stage of development, highlighting the importance of continued empirical evaluation as AI technologies evolve. Future research will be essential to further clarify effectiveness, sustainability, and ethical considerations, particularly through longitudinal and interdisciplinary studies. Overall, the findings support AI as an emerging, context-sensitive tool in nursing, with the capacity to contribute to high-quality, human-centered care when implemented responsibly.
